# Multi-cohort evidence linking edentulism to frailty among older adults

**DOI:** 10.1038/s41598-025-27516-6

**Published:** 2025-12-15

**Authors:** Jun He, Xun Xie, Ying Wang

**Affiliations:** 1 Department of Dentistry, Sir Run Run Shaw Hospital, Zhejiang University School of Medicine, Hangzhou, 310000 China; 2 School of Stomatology, Zhejiang University School of Medicine, Hangzhou, 310000 China; 3 Provincial Clinical Research Center for Oral Diseases, Key Laboratory of Oral Biomedical Research of Zhejiang Province, Cancer Center of Zhejiang University, Engineering Research Center of Oral Biomaterials and Devices of Zhejiang Province, Hangzhou, 310000 China

**Keywords:** Edentulism, Frailty, Older adults, Multi-cohort, Diseases, Health care, Medical research, Risk factors

## Abstract

To investigate the association between edentulism and frailty across nationally representative cohorts in China, the United Kingdom, and the United States. A harmonized analysis was conducted using longitudinal data from the CHARLS, ELSA, and HRS cohorts. This study examined the association between self-reported edentulism (exposure) and frailty (outcome), with adjustment for self-reported covariates including age, gender, lifestyle factors, and comorbidities. Multivariable linear and logistic regression models were applied to examine the associations of edentulism with the frailty index and frailty phenotype, respectively. To synthesize effect estimates across cohorts, fixed- and random-effects meta-analyses were conducted. Robustness was assessed through stratified analyses by age and gender, as well as through multiple imputation to address missing data. A total of 9,869 participants from CHARLS (female: 52.9%, mean age: 61.2 years), 5,083 from ELSA (female: 55.7%, mean age: 62.7 years), and 12,322 from HRS (female: 59.5%, mean age: 65.8 years) were included. Meta-analysis of the fully adjusted models across cohorts revealed that edentulous individuals exhibited significantly higher frailty index scores (pooled mean difference = 2.68; 95% confidence interval [CI]: 1.67–3.69) and increased odds of frailty status (pooled odds ratio = 1.38; 95% CI: 1.26–1.50) compared to dentate counterparts. These associations remained robust in stratified and sensitivity analyses. Edentulism is independently associated with frailty across aging populations. These findings underscore the clinical relevance of oral health in geriatric risk assessment and support the integration of dental evaluation into multidisciplinary strategies for frailty management.

**Supplementary Information:**

The online version contains supplementary material available at 10.1038/s41598-025-27516-6.

## Introduction

As human life expectancy continues to rise, population aging is accelerating worldwide, placing growing strain on healthcare systems and social infrastructure^1^. Frailty, a progressive clinical syndrome characterized by diminished physiological reserve and resilience, substantially increases the risk of falls, disability, hospitalization, and mortality among older adults^2^. Importantly, frailty is also recognized as a dynamic and potentially reversible condition, highlighting the critical importance of identifying modifiable risk factors to inform prevention and intervention strategies^3^. Given its significant clinical and societal burden, increasing attention is being directed toward identifying modifiable risk factors to inform prevention strategies^4^.

Edentulism, defined as the complete loss of all natural teeth, affects millions of older adults, but remains insufficiently acknowledged within the discourse of geriatric health^5^. Beyond its immediate impact on oral function, edentulism compromises masticatory efficiency, restricts dietary diversity, and contributes to malnutrition, systemic inflammation, and physical deterioration^6^. These multifaceted consequences position edentulism as a salient yet frequently overlooked indicator of systemic physiological decline in the elderly^7^.

The persistent belief that “tooth loss is an unavoidable aspect of aging” continues to influence public perception. However, this notion is increasingly at odds with a growing body of evidence emphasizing the integral role of oral health in overall well-being^8^. Edentulism should not be regarded as an inevitable outcome of aging but rather as the culmination of cumulative biological, behavioral, and social vulnerabilities^9^. As the concept of the oral-systemic connection gains traction within geriatric medicine, tooth loss is being reconceptualized not merely as a localized dental issue but as both a visible manifestation and a potential contributor to multisystem deterioration, including the development of frailty^10^.

A recent meta-analysis demonstrated that edentulous older adults are approximately 69% more likely to be frail compared to their dentate counterparts^11^. Existing aggregate evidence relies predominantly on summary-level meta-analyses of heterogeneous investigations. Variations in population characteristics, variable definitions, and analytical methods across original studies constrain the credibility and generalizability of the reported associations. To address this gap, the present study investigates the association between edentulism and frailty using harmonized analytical procedures across three large-scale, previously unexamined, population-based cohorts.

## Materials and methods

### Study design and population

We conducted a longitudinal analysis utilizing the most recent available wave of data from three nationally representative cohort studies: the China Health and Retirement Longitudinal Study (CHARLS; waves 1–4, 2011–2018), the English Longitudinal Study of Ageing (ELSA; waves 7–9, 2014–2019), and the Health and Retirement Study (HRS; waves 11–14, 2012–2018), conducted in China, the United Kingdom, and the United States, respectively^12–14^. The follow-up waves were intentionally chosen to maximize the comparability of the time span across cohorts and to ensure data completeness for a robust comparative analysis. Ethical approvals were obtained from the Institutional Review Board of Peking University (CHARLS), the London Multi-Centre Research Ethics Committee (ELSA), and the University of Michigan (HRS). All participants provided written informed consent. Standardized exclusion criteria across the cohorts included: (1) age ≤ 50 years at baseline; and (2) missing data on edentulism, frailty, or any covariates.

### Edentulism assessment

Edentulism was defined as the self-reported complete loss of all natural teeth, assessed through a structured questionnaire administered during the designated baseline wave for this study (CHARLS: wave 1, 2011; ELSA: wave 7, 2014; HRS: wave 11, 2012). Participants responded to a standardized and validated screening item: “Have you experienced complete loss of all natural teeth?” Responses were recorded as a dichotomous variable (yes/no).

### Frailty assessment

The frailty index (FI) was constructed using 32 age-related variables encompassing chronic diseases, functional impairments, self-rated health, depressive symptoms, and cognitive function^15^. Items 1–31 were coded as binary variables, while Item 32 (cognitive score) was continuous (Supplementary Table 1). The FI was calculated as the ratio of present deficits to the total number of non-missing items, producing a score ranging from 0 to 100%, with higher scores indicating greater frailty. Based on established thresholds, participants were classified as frail (FI > 25%) or robust (FI ≤ 25%)^16^. Individuals with more than two missing items were excluded. Due to substantial missing data at baseline, the most recent wave with complete frailty information was used (CHARLS: wave 4, 2018; ELSA: wave 9, 2019; HRS: wave14, 2018).

### Covariates

All covariates were ascertained from the baseline wave of each cohort: CHARLS, wave 1, 2011; ELSA, wave 7, 2014; HRS, wave 11, 2012. Baseline covariates included sociodemographic characteristics (age, gender, ethnicity, educational attainment), lifestyle factors (smoking status, drinking status, physical activity), and clinical conditions (hypertension, diabetes). To ensure cross-cohort comparability, ethnicity was dichotomized as majority (Han ethnicity in CHARLS; White ethnicity in ELSA and HRS) versus non-majority. Educational attainment was categorized as below high school, high school, and college or above. Smoking and drinking status were each classified as never or ever. Physical activity was defined as engagement in regular moderate or vigorous exercise. Hypertension and diabetes were identified based on self-reported physician diagnoses.

### Statistical analyses

Descriptive statistics were used to summarize baseline characteristics. Continuous variables were presented as means with standard deviations (SD), and categorical variables as frequencies with percentages. Group differences across edentulism status were assessed using analysis of variance (ANOVA) for continuous variables and chi-square tests for categorical variables.

Associations between edentulism and frailty outcomes were evaluated using multivariable linear regression for the frailty index and multivariable logistic regression for the frailty status. Effect estimates were expressed as mean differences (MDs) or odds ratios (ORs) with corresponding 95% confidence intervals (CIs). Four sequential models were constructed: Model 1 was unadjusted; Model 2 adjusted for age, gender, ethnicity, and education; Model 3 further adjusted for smoking status, alcohol status, and physical activity; and Model 4 additionally included hypertension and diabetes.

To synthesize effect estimates across the three cohorts, both fixed- and random-effects meta-analyses were conducted. Heterogeneity was assessed using the I^2^ statistic; when I^2^ exceeded 50%, random-effects models were applied, otherwise fixed-effects models were used. Robustness was evaluated through stratified analyses by age and gender, and through multiple imputation for missing data using the missForest and Amelia algorithms.

All analyses were performed using R software (version 4.3.2; R Foundation for Statistical Computing, Vienna, Austria). The software is freely available at https://www.R-project.org/. A two-sided p-value < 0.05 was considered statistically significant.

## Results

**Fig. 1:**
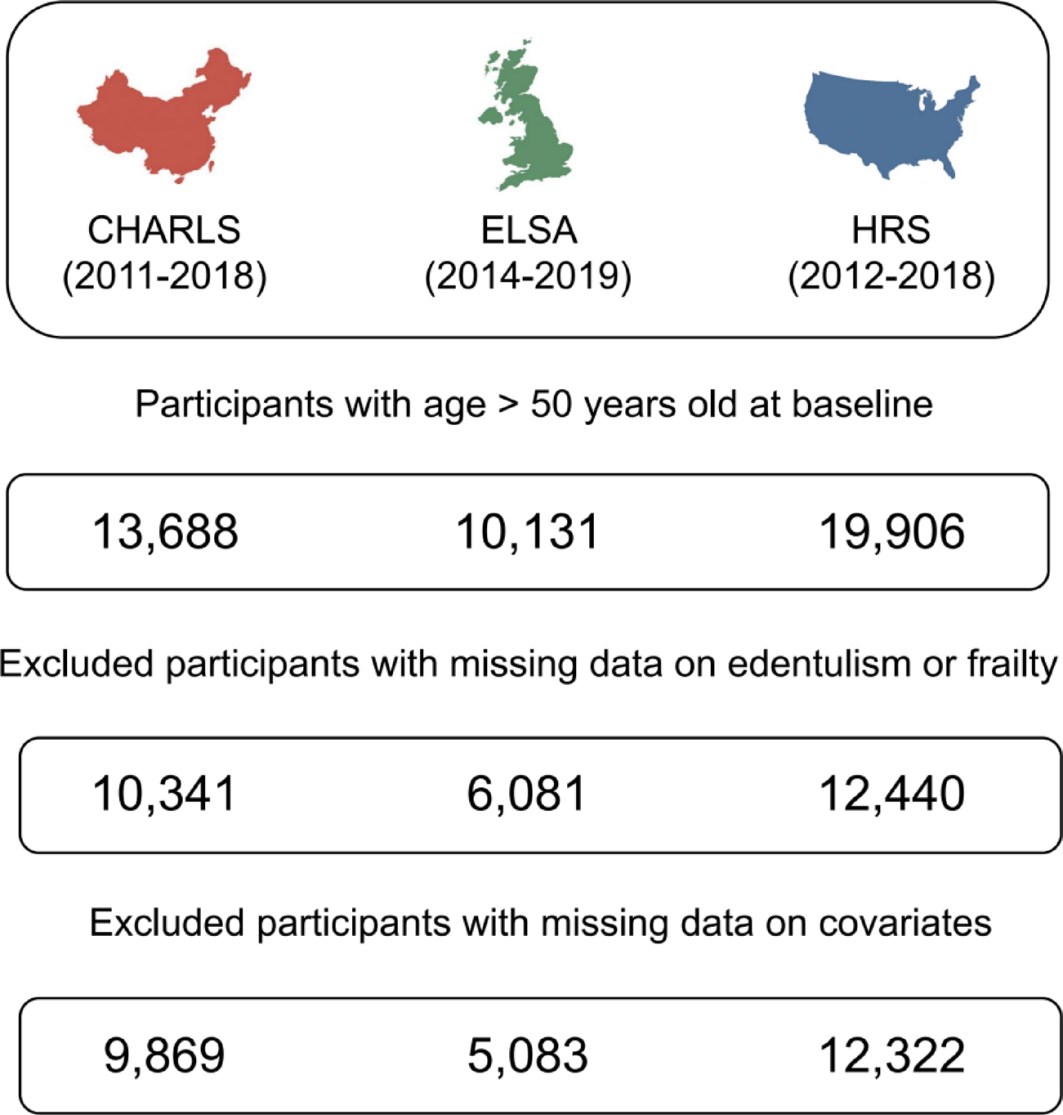
Flowchart of participant selection.

According to the exclusion criteria, a total of 9,869 participants from CHARLS (52.9% female; mean age, 61.2 years), 5,083 from ELSA (55.7% female; mean age, 62.7 years), and 12,322 from HRS (59.5% female; mean age, 65.8 years) were included in the final analysis (Fig. 1).

Baseline characteristics of participants by edentulism status across cohorts are summarized in Table 1. In CHARLS, 9.17% of participants were edentulous; in ELSA, 8.42%; and in HRS, 14.62%. Across all three cohorts, edentulous individuals were significantly older and had higher frailty index scores compared to dentate participants. They were also more likely to be female, have lower educational attainment (e.g., below high school), and report never drinking alcohol. Additionally, edentulous participants had a higher prevalence of hypertension and diabetes in ELSA and HRS, though this difference was not statistically significant in CHARLS. Conversely, dentate individuals were more likely to engage in regular physical activity and had a lower prevalence of frailty. These patterns highlight consistent sociodemographic and health disparities associated with edentulism across diverse populations.

**Table 1:** Characteristics of included participants.

Characteristic	CHARLS (*N* = 9,869)		*P* value	ELSA (*N* = 5,083)		*P* value	HRS (*N* = 12,322)		*P* value
	Dentate	Edentulism		Dentate	Edentulism		Dentate	Edentulism	
Number	8964 (90.83)	905 (9.17)		4655 (91.58)	428 (8.42)		10,521 (85.38)	1801 (14.62)	
Age (mean ± SD)	60.59 (7.25)	67.62 (8.42)	< 0.001	65.21 (7.64)	71.18 (8.14)	< 0.001	65.28 (9.46)	68.70 (9.86)	< 0.001
Frailty index (mean ± SD)	17.12 (11.47)	21.79 (13.37)	< 0.001	15.21 (11.76)	22.76 (14.54)	< 0.001	18.52 (13.23)	26.06 (15.68)	< 0.001
Gender, n (%)			< 0.001			< 0.001			0.001
Female	4685 (52.3)	536 (59.2)		2555 (54.9)	275 (64.3)		6200 (58.9)	1136 (63.1)	
Male	4279 (47.7)	369 (40.8)		2100 (45.1)	153 (35.7)		4321 (41.1)	665 (36.9)	
Ethnicity, n (%)*			0.155			0.478			< 0.001
Majority	8304 (92.6)	826 (91.3)		4536 (97.4)	420 (98.1)		7621 (72.4)	1138 (63.2)	
Minority	660 (7.4)	79 (8.7)		119 (2.6)	8 (1.9)		2900 (27.6)	663 (36.8)	
Education, n (%)			< 0.001			< 0.001			< 0.001
Below high school	8005 (89.3)	856 (94.6)		1052 (22.6)	226 (52.8)		1483 (14.1)	597 (33.1)	
High school	840 (9.4)	43 (4.8)		2531 (54.4)	176 (41.1)		6122 (58.2)	1085 (60.2)	
College or above	119 (1.3)	6 (0.7)		1072 (23.0)	26 (6.1)		2916 (27.7)	119 (6.6)	
Smoking status, n (%)			0.849			< 0.001			< 0.001
Never	6237 (69.6)	633 (69.9)		4182 (89.8)	347 (81.1)		9293 (88.3)	1380 (76.6)	
Ever	2727 (30.4)	272 (30.1)		473 (10.2)	81 (18.9)		1228 (11.7)	421 (23.4)	
Drinking status, n (%)			< 0.001			< 0.001			< 0.001
Never	6016 (67.1)	666 (73.6)		1388 (29.8)	213 (49.8)		4163 (39.6)	1070 (59.4)	
Ever	2948 (32.9)	239 (26.4)		3267 (70.2)	215 (50.2)		6358 (60.4)	731 (40.6)	
Physical activity, n (%)			< 0.001			< 0.001			< 0.001
Regular	4005 (44.7)	314 (34.7)		3193 (68.6)	220 (51.4)		7949 (75.6)	1107 (61.5)	
Irregular	4959 (55.3)	591 (65.3)		1462 (31.4)	208 (48.6)		2572 (24.4)	694 (38.5)	
Hypertension, n (%)			0.876			< 0.001			< 0.001
No	6515 (72.7)	655 (72.4)		2918 (62.7)	208 (48.6)		4646 (44.2)	618 (34.3)	
Yes	2449 (27.3)	250 (27.6)		1737 (37.3)	220 (51.4)		5875 (55.8)	1183 (65.7)	
Diabetes, n (%)			0.887			< 0.001			< 0.001
No	8403 (93.7)	850 (93.9)		4297 (92.3)	372 (86.9)		8336 (79.2)	1267 (70.3)	
Yes	561 (6.3)	55 (6.1)		358 (7.7)	56 (13.1)		2185 (20.8)	534 (29.7)	
Frailty, n (%)			< 0.001			< 0.001			< 0.001
No	5577 (62.2)	423 (46.7)		3567 (76.6)	226 (52.8)		6615 (62.9)	703 (39.0)	
Yes	3387 (37.8)	482 (53.3)		1088 (23.4)	202 (47.2)		3906 (37.1)	1098 (61.0)	

*The majority group is defined as Han ethnicity in CHARLS, and as White ethnicity in both ELSA and HRS.

**Fig. 2:**
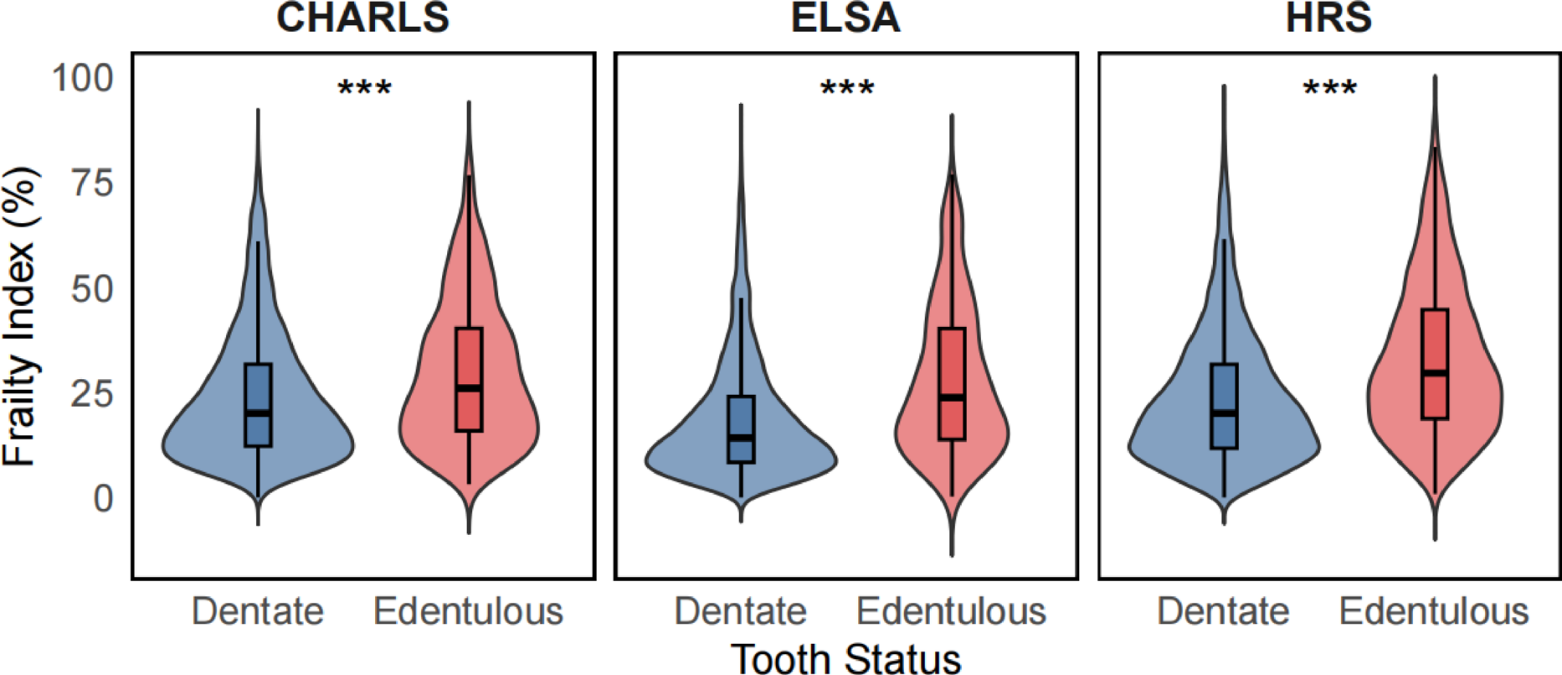
Violin plots illustrating the distribution of follow-up frailty index by tooth status across three population-based cohorts. ***p-value < 0.001.

In the CHARLS, ELSA, and HRS cohorts, the frailty index of edentulous individuals was significantly higher than that of dentate individuals (Fig. 2).

In unadjusted models (Model 1), edentulous individuals were significantly more likely to be frail than their dentate counterparts, with ORs of 1.88 (95% CI: 1.64–2.15) in CHARLS, 2.93 (95% CI: 2.39–3.58) in ELSA, and 2.65 (95% CI: 2.39–2.93) in HRS. Edentulism was also associated with higher FI scores, with MDs of 5.66% (95% CI: 4.59%–6.72%), 9.49% (95% CI: 8.07%–10.91%), and 9.58% (95% CI: 8.78%–10.38%) in CHARLS, ELSA, and HRS, respectively.

In the fully adjusted model (Model 2), edentulous individuals had increased odds of frailty, with ORs of 1.24 (95% CI: 1.06–1.44) in CHARLS, 1.33 (95% CI: 1.05–1.68) in ELSA, and 1.48 (95% CI: 1.31–1.67) in HRS. Correspondingly, FI scores remained elevated by 1.82% (95% CI: 0.81%−2.84%) in CHARLS, 2.66% (95% CI: 1.39%−3.94%) in ELSA, and 3.40% (95% CI: 2.70%−4.10%) in HRS (Table 2).

**Table 2:** Association between edentulism and frailty.

Study	Model 1		Model 2	
	OR (95% CI)	*P* value	OR (95% CI)	*P* value
CHARLS (*N* = 9,869)				
Frailty status (binary)	1.88 (1.64–2.15)	2.77 × 10^− 19^	1.24 (1.06–1.44)	0.006
Frailty index (per 1% unit)	5.66 (4.59–6.72)	3.07 × 10^− 25^	1.82 (0.81–2.84)	4.26 × 10^− 4^
ELSA (*N* = 5,083)				
Frailty status (binary)	2.93 (2.39–3.58)	1.39 × 10^− 25^	1.33 (1.05–1.68)	0.016
Frailty index (per 1% unit)	9.49 (8.07–10.91)	1.09 × 10^− 38^	2.66 (1.39–3.94)	4.16 × 10^− 5^
HRS (*N* = 12,322)				
Frailty status (binary)	2.65 (2.39–2.93)	4.55 × 10^− 77^	1.48 (1.31–1.67)	9.86 × 10^− 11^
Frailty index (per 1% unit)	9.58 (8.78–10.38)	1.25 × 10^− 119^	3.4 (2.7–4.1)	2.66 × 10^− 21^

Model 1: unadjusted.

Model 2: adjust for: age, gender, ethnicity, education, smoking status, drinking status, physical activity, hypertension, and diabetes.

Note: Exposure and covariates were assessed at baseline (CHARLS-2011, ELSA-2014, HRS-2012), and frailty outcomes were derived from the follow-up wave (CHARLS-2018, ELSA-2019, HRS-2018).

**Fig. 3:**
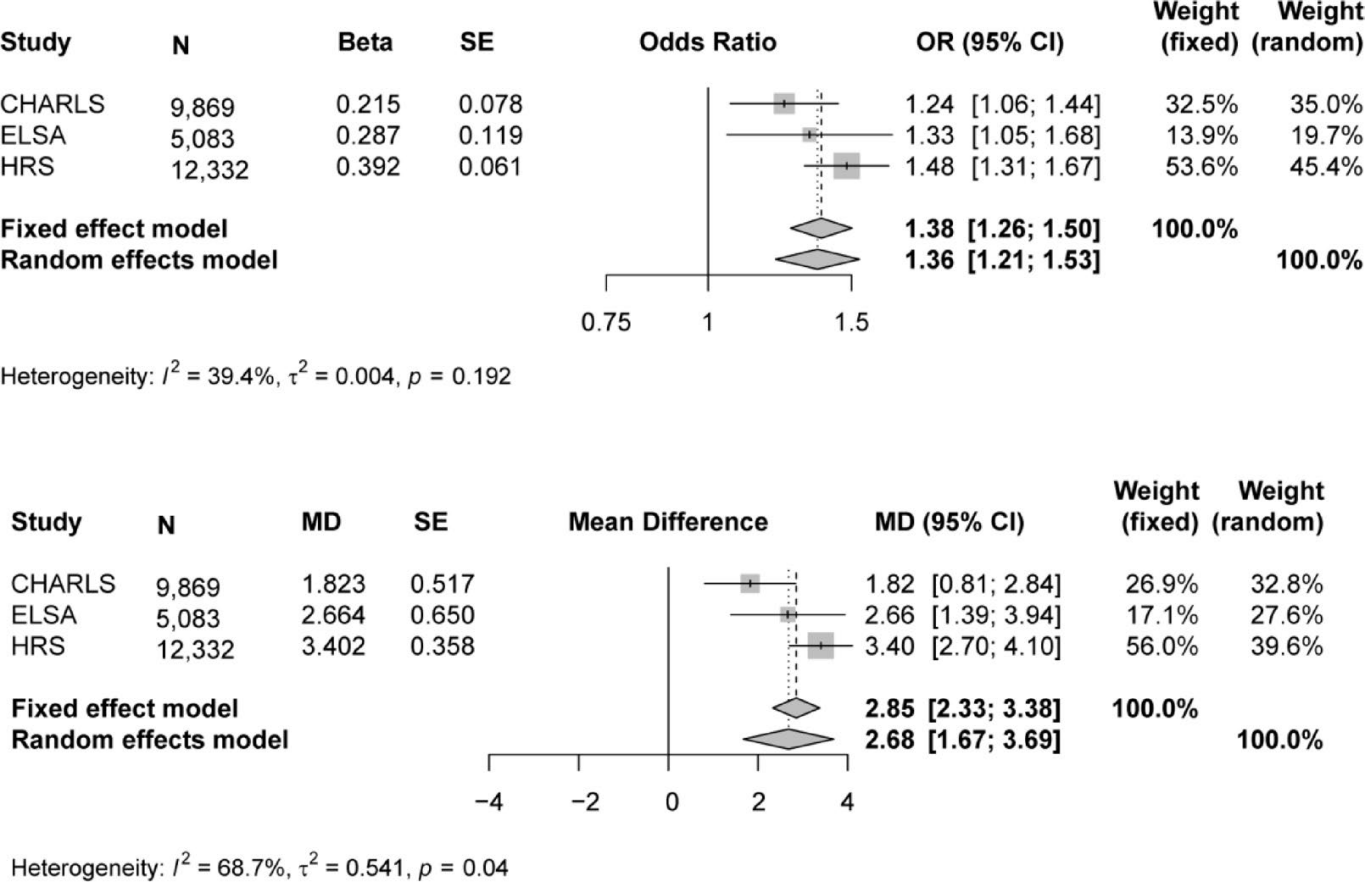
Meta-analysis of the association between edentulism and frailty across three cohorts.

To synthesize effect estimates and enhance statistical power, meta-analyses were conducted across the three cohorts. For the frailty phenotype, low heterogeneity was observed (I^2^ = 39.4%), and a fixed-effects model yielded a pooled OR of 1.38 (95% CI: 1.26–1.50), indicating significantly greater odds of frailty among edentulous individuals. In contrast, substantial heterogeneity was observed for FI scores (I^2^ = 68.7%); using a random-effects model, edentulism was associated with a pooled MD of 2.68 (95% CI: 1.67–3.69) in FI scores compared to dentate counterparts (Fig. 3).

**Fig. 4:**
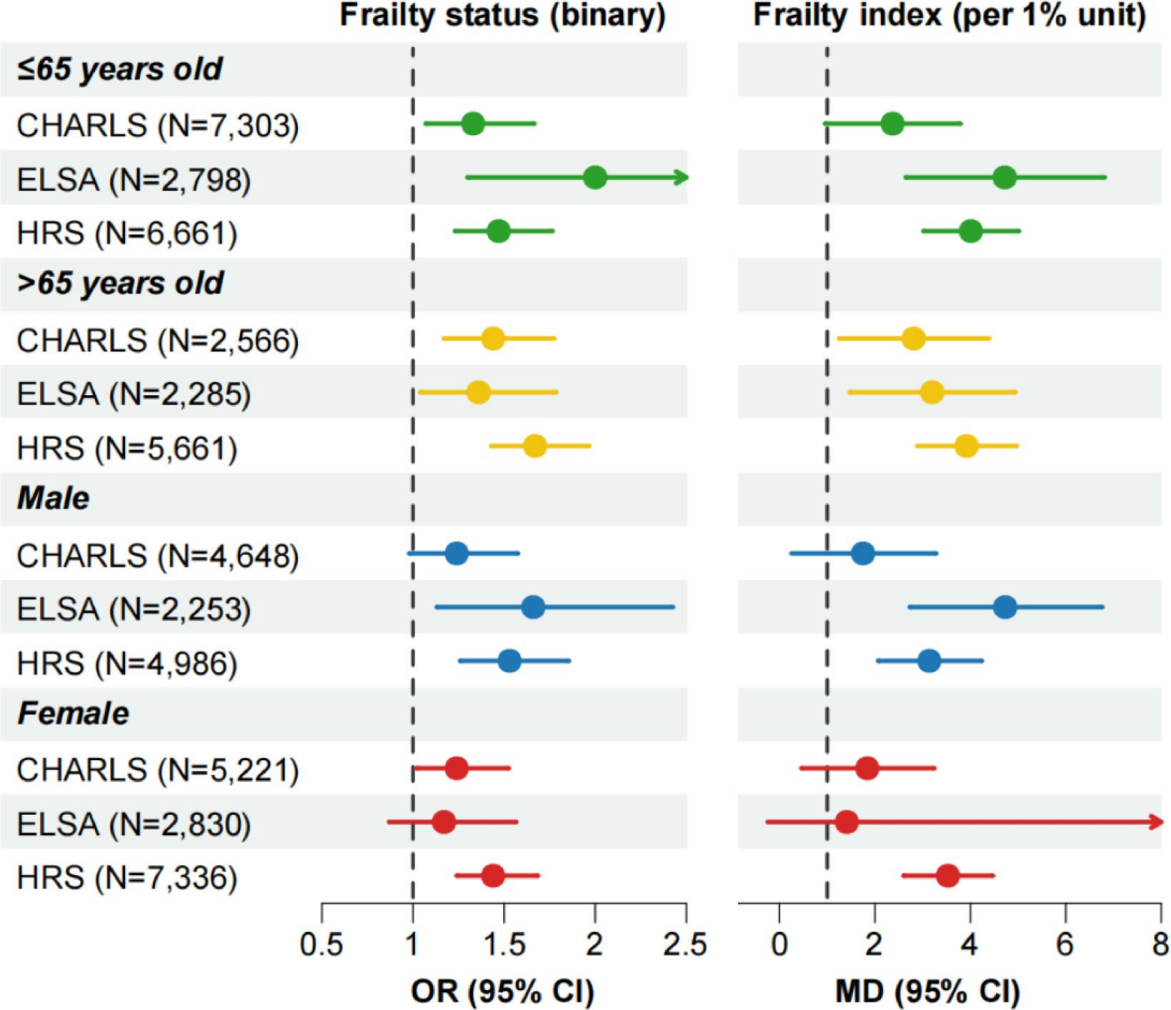
Stratified analyses of the edentulism-frailty association by age and gender.

In age-stratified analyses, significant heterogeneity was observed in the ELSA cohort, where the association between edentulism and frailty status was stronger among participants aged ≤ 65 years (Supplementary Table 2). Nevertheless, age-stratified associations remained statistically significant across all cohorts (Fig. 4, Supplementary Table 3). Gender-stratified analyses didn’t reveal consistent heterogeneity; however, in the fully adjusted model (Model 4) for ELSA, the association between edentulism and frailty outcomes was no longer statistically significant among female participants (Fig. 3, Supplementary Table 4).

To address potential bias from missing data, multiple imputation was performed using both the missForest and Amelia algorithms. Post-imputation analyses confirmed the robustness of the findings, with consistent associations observed between edentulism and frailty outcomes across all three cohorts (Supplementary Table 5). When analyzing only the baseline non-frail population, edentulism remained significantly associated with incident frailty in HRS after full adjustment, but showed attenuated associations in CHARLS and ELSA cohorts (Supplementary Table 6). Also, after adjusting for baseline frailty index, the association between edentulism and frailty was substantially attenuated and became non-significant in CHARLS and ELSA cohorts, but remained significant in HRS. (Supplementary Table 7).

## Discussion

Using harmonized data from nationally representative cohorts in China, the United Kingdom, and the United States, this study provides compelling evidence that edentulism is independently associated with frailty in older adults, reinforcing the critical and generalizable role of oral health in the context of geriatric vulnerability.

Our findings consolidate and extend epidemiological evidence linking tooth loss to frailty. Single-nation studies across diverse populations, including China (CLHLS), Chile, and the UK, have consistently demonstrated elevated frailty risk associated with edentulism and partial tooth loss^16–18^. This association has been further corroborated by recent meta-analyses, which synthesized global evidence and confirmed that tooth loss, functional dentition absence, and lack of denture use significantly predict frailty^11,19,20^.

However, these previous investigations have been constrained by methodological heterogeneity, inconsistent frailty definitions, and limited generalizability^21^. To address these limitations, recent research has shifted toward harmonized, multi-cohort designs, as seen in studies using CHARLS, ELSA, HRS, SHARE, and MHAS^22–24^, to improve cross-national comparability and analytical robustness. This harmonized multinational approach strengthens the evidence for a potential causal relationship by eliminating methodological heterogeneity through standardized protocols. It ensures external validity by replicating findings across disparate populations, and establishes cross-cultural generalizability by demonstrating consistent effects despite varying socioeconomic contexts, healthcare systems, and cultural norms.

Following this paradigm, our study integrates data from three independent, methodologically harmonized cohorts. Our approach provides the first directly comparable evidence across three diverse national contexts (China, the UK, and the US), demonstrating that the edentulism-frailty link is robust and persists irrespective of major differences in socioeconomic contexts, healthcare systems, and cultural norms (including diet and aging cultures). By utilizing standardized models, stratified analyses, and rigorous sensitivity testing, we further establish that this relationship is not attributable to methodological artifact. Consequently, our findings substantially extend knowledge by confirming the universal nature of this association and providing a methodologically robust, generalizable evidence base for future research and policy.

The biological plausibility of the association between edentulism and frailty is supported by multiple converging pathways. Tooth loss may contribute to frailty through intertwined nutritional, psychosocial, and functional mechanisms that collectively accelerate physiological decline^25^. Impaired mastication restricts the intake of high-quality protein, fiber, and essential micronutrients that are critical components for maintaining muscle mass, immune function, and metabolic homeostasis^26^. Chronic nutritional deficiencies may subsequently lead to sarcopenia, energy imbalance, and immune dysregulation, all of which are hallmark features of frailty in later life^27^. Besides, the psychosocial consequences of edentulism are increasingly recognized. Aesthetic concerns, reduced self-esteem, and communication difficulties may contribute to social withdrawal and depressive symptoms—both of which are independently associated with elevated frailty risk^28^. These findings suggest that although tooth loss is anatomically localized, it may instigate systemic vulnerability through behavioral disengagement and biological dysregulation^29^.

Inflammatory and neurophysiological pathways further support the role of poor oral health in frailty development^30^. Periodontitis, the leading cause of adult tooth loss, triggers chronic low-grade inflammation, marked by elevated levels of interleukin-6 (IL-6) and C-reactive protein (CRP), two biomarkers consistently linked to frailty, sarcopenia, and functional impairment^31^. Additionally, the loss of periodontal proprioceptors may compromise neuromuscular feedback essential for postural control, thereby increasing the risk of falls, a sentinel event in frailty progression^32^. These mechanisms are further exacerbated by comorbid conditions such as diabetes and cardiovascular disease, which amplify systemic inflammation and accelerate physical decline, collectively supporting a syndemic framework in which tooth loss serves both as a marker of underlying vulnerability and an active contributor to the onset and progression of frailty^33^.

It is important to note the substantial baseline differences in demographics and health status among CHARLS, ELSA, and HRS (Table 1), which reflect real-world disparities in aging experiences, healthcare systems, and socioeconomic contexts across China, the UK, and the US. Rather than undermining our findings, this diversity enhances their external validity. These population differences likely contributed to the substantial heterogeneity (I² > 60%) in the frailty index meta-analysis. Rather than a statistical limitation, this heterogeneity probably reflects genuine contextual variation in the association’s strength. Importantly, despite variations in baseline characteristics and effect size, the consistent direction of effect across all cohorts underscores a robust, transcultural relationship between edentulism and frailty. Furthermore, the heterogeneity observed in stratified analyses, such as the non-significant association among females in ELSA, suggests potential effect modification by unmeasured country- or gender-specific factors, such as denture quality or healthcare access^34^. These findings highlight the need to investigate social and clinical determinants underlying this varied risk.

Despite its strengths, our study has several limitations. First, due to variation in data availability across cohorts, we were unable to incorporate additional oral health indicators, such as the number of remaining teeth or denture use, into a unified analytical model. The use of a binary edentulism variable may therefore overlook clinically meaningful variation. For instance, well-fitted dentures might reduce frailty risk by improving nutrition, while poor oral health among dentate individuals could increase risk due to inflammation. So, the observed association likely reflects both historical inflammatory exposure and ongoing nutritional influences; however, distinguishing their relative contributions requires finer-grained data. Second, although our design incorporated a prospective component, with edentulism assessed at baseline and frailty measured at follow-up, the lack of individualized frailty trajectories limited our ability to conduct time-to-event analyses (e.g., Cox regression). Therefore, the strong association observed does not resolve issues of temporality, and a bidirectional relationship remains plausible. Third, while our sensitivity analyses addressed key confounding by baseline health status, residual confounding from unmeasured factors (e.g., dietary biomarkers, inflammatory mediators, and oral hygiene practices) may partially explain the attenuated associations observed after adjusting for baseline frailty and the inconsistencies across cohorts. This underscores the complex, multifactorial nature of the edentulism-frailty relationship. Finally, we relied on self-reported edentulism status. Although this measure has been validated in large-scale epidemiological studies due to its high specificity and acceptable sensitivity^35,36^, and is practically necessary in such population-based studies, it may still introduce misclassification bias^37^.

Despite these limitations, our findings highlight key clinical implications. While edentulism is irreversible, its sequelae can be managed. Clinicians should address inflammatory sources (e.g., residual roots)^38^ and provide functional dentures to restore mastication, improve nutrition, and enhance quality of life^39^. Edentulism should thus trigger comprehensive oral rehabilitation rather than signify an endpoint. Future research should incorporate multiple dimensions of oral health indicators, such as oral frailty, chewing ability and oral hygiene, in order to determine precise intervention targets and thereby alleviate the condition of frailty.

## Conclusion

In conclusion, this multi-cohort analysis provides robust evidence that edentulism is independently associated with frailty in older adults. These findings underscore the role of oral health as a modifiable and clinically relevant factor within the multidimensional construct of frailty. From both clinical and public health perspectives, integrating dental assessments into routine geriatric evaluations may enable the early identification of at-risk individuals and inform more comprehensive frailty prevention strategies. Future research should clarify the causal mechanisms and biological mediators of this association, and assess the efficacy of targeted oral health interventions in modifying the course of frailty.

## Supplementary Information

Below is the link to the electronic supplementary material.

Supplementary Material 1

## Data Availability

The original datasets can also be accessed upon application through the official websites of the respective cohort studies. CHARLS (China Health and Retirement Longitudinal Study): http://charls.pku.edu.cn. ELSA (English Longitudinal Study of Ageing): https://www.elsa-project.ac.uk. HRS (Health and Retirement Study): https://hrs.isr.umich.edu.
